# Assessment of risk factors of lymph node metastasis and prognosis of Siewert II/III adenocarcinoma of esophagogastric junction: A retrospective study

**DOI:** 10.1097/MD.0000000000037289

**Published:** 2024-03-01

**Authors:** Yidong Huang, Zhi Zheng, Rui Xu, Haiqiao Zhang, Jie Yin, Xiaoye Liu, Jun Zhang, Guangyong Chen, Zhongtao Zhang

**Affiliations:** a Department of General Surgery, Beijing Friendship Hospital, Capital Medical University, Beijing, China; b Beijing Key Laboratory of Cancer Invasion and Metastasis Research & National Clinical Research Center for Digestive Diseases, Xi-Cheng District, Beijing, China; c Department of Pathology, Beijing Friendship Hospital, Capital Medical University, Beijing, China.

**Keywords:** adenocarcinoma of esophagogastric junction, lymphatic metastasis, risk factor, Siewert II/III, survival analysis

## Abstract

Adenocarcinoma of the esophagogastric junction (AEG) has a high incidence, and the extent of lymph node dissection (LND) and its impact on prognosis remain controversial. This study aimed to explore the risk factors for lymph node metastasis (LNM) and prognosis in Siewert II/III AEG patients. A retrospective review of 239 Siewert II/III AEG patients surgically treated at Beijing Friendship Hospital from July 2013 to December 2022 was conducted. Preoperative staging was conducted via endoscopy, ultrasound gastroscopy, CT, and biopsy. Depending on the stage, patients received radical gastrectomy with LND and chemotherapy. Clinicopathological data were collected, and survival was monitored semiannually until November 2023. Utilizing logistic regression for data analysis and Cox regression for survival studies, multivariate analysis identified infiltration depth (OR = 0.038, 95% CI: 0.011–0.139, *P* < .001), tumor deposit (OR = 0.101, 95% CI: 0.011–0.904, *P* = .040), and intravascular cancer embolus (OR = 0.234, 95% CI: 0.108–0.507, *P* < .001) as independent predictors of LNM. Lymph nodes No. 1, 2, 3, 4, 7, 10, and 11 were more prone to metastasis in the abdominal cavity. Notably, Siewert III AEG patients showed a higher metastatic rate in nodes No. 5 and No. 6 compared to Siewert II. Mediastinal LNM was predominantly found in nodes No. 110 and No. 111 for Siewert II AEG, with rates of 5.45% and 3.64%, respectively. A 3-year survival analysis underscored LNM as a significant prognostic factor (*P* = .001). Siewert II AEG patients should undergo removal of both celiac and mediastinal lymph nodes, specifically nodes No. 1, 2, 3, 4, 7, 10, 11, 110, and 111. Dissection of nodes No. 5 and No. 6 is not indicated for these patients. In contrast, Siewert III AEG patients do not require mediastinal LND, but pyloric lymphadenectomy for nodes No. 5 and No. 6 is essential. The presence of LNM is associated with poorer long-term prognosis. Perioperative chemotherapy may offer a survival advantage for AEG patients.

## 1. Introduction

Adenocarcinoma of esophagogastric junction (AEG) is a type of adenocarcinoma lesion that originates from the glandular epithelium of the gastrointestinal tract and invades esophagogastric junction.^[1]^ In recent years, the incidence and mortality of AEG have increased.^[2–4]^ This region is a special anatomical location, involving complex operation and multiple lymphatic drainage approaches.^[5]^ The drainage boundary and dissection extends from the lymph nodes around the stomach and it is localized around the cardia for multiple groups of lymph nodes.^[6]^ As a result, AEG can be treated with diverse surgical methods and exhibits a worse prognosis compared with common gastric cancer.^[7,8]^ Previous studies have shown that mediastinal lymph node metastasis (LNM) accounts for 10% to 20% of all lymphatic metastasis in AEG.^[9–11]^ However, other studies reported that the mediastinal LNM rate was low.^[12,13]^ Therefore, it is urgent to confirm whether the mediastinal lymph node dissection (LND) of the lymph node drainage areas is required in Siewert II/III AEG in order to improve the curative effect of radical operation and prognosis. The present study attempted to analyze the risk factors of LNM and the follow-up survival data in patients with Siewert II/III AEG in order to provide more accurate treatments for AEG patients with different clinicopathological features.

## 2. Methods

### 2.1. General information

The clinical data from 239 Siewert II/III AEG patients undergoing surgical treatment (open or laparoscopic surgery) were selected between July 2013 and Dec 2022 from the General Surgery Department, Beijing Friendship Hospital. The 239 cases were retrospectively studied. Before surgery, Siewert II/III type AEG patients underwent routine endoscopy, ultrasound gastroscopy, and enhanced CT scans of the abdomen and pelvis to assess tumor infiltration depth and lymphatic and distant metastasis. Pathological biopsy was also performed for diagnosis, as it is considered the gold standard for cancer detection. The early-stage AEG patients (cT_1_) received radical gastrectomy initially and no <15 lymph nodes were dissected.^[14,15]^ For the lymph node number, the 8^th^ version AJCC/UICC TNM staging system for gastric cancer was adopted, which recommends removing at least 15 lymph nodes for accurate staging.^[16]^ Subsequently, certain patients (pT_1_N_1-3_M_0_) would receive first-line chemotherapy regimen (XELOX). In addition, the patients with advanced stage AEG (cT_2-4_N_0-3_M_0_) were confirmed with postoperative pathological examination (pT_2-4_N_0-3_M_0_) and received first-line chemotherapy regimen (XELOX) following surgery. For advanced-stage patients (cT_3-4_N_+_M_0_) who could not achieve R0 resection, 3-cycle preoperative neoadjuvant chemotherapy (ECF) was initially administered. Subsequently, the patients would receive surgery if complete tumor resection was scheduled. Following operation, they would also receive postoperative chemotherapy (XELOX). The present study gained approval from the Ethics committee of the Beijing Friendship Hospital (approval number 2016-P2-039-02). The requirement for individual consent was waived due to the retrospective nature of the study.

### 2.2. Inclusion criteria

Patients who were diagnosed as Siewert type II/III AEG (epicenter of the tumor located between 1 cm above to 5 cm below the esophagogastric junction) by pathological biopsy and endoscopy were included in the study. The patients were evaluated based on the clinical stage and the epicenter of the tumor prior to the surgery, including abdominal enhanced CT and/or ultrasound gastroscopy. The clinical stage was T_1-4_N_0-3_M_0_. Tumor staging was assessed as follows: Invasion of the tumor into the esophagogastric junction and location of its center at least 2 cm below the esophagogastric junction or invasion of the tumor into the esophagogastric junction and location of its center within 2 cm below the esophagogastric junction. For any of the aforementioned cases, the 8^th^ version AJCC/UICC TNM staging system for gastric cancer was adopted. If the tumor invaded the esophagogastric junction and its center was located within 2 cm below the esophagogastric junction, then the 8^th^ version of the AJCC/UICC TNM staging system for esophageal cancer was adopted.^[17]^ The patients who had a physical fitness score, ECOG ≤ 2 points and were tolerant to radical gastrectomy were also included in the selection process. Patients who did not have a previous history of gastrointestinal operation, chemotherapy or radiotherapy were included in the study.

### 2.3. Exclusion criteria

The exclusion criteria used in the present study were the following: Patients who suffered from gastric remnant carcinoma, recurrent cancer of gastric remnant or multiple primary malignant neoplasm of pelvis or had malignancy history within 5 years. Patients who were unwilling to receive operations. Patients who were confirmed with metastatic gastric cancer or gastric cancer of other parts intraoperatively. Patients who suffered from uncontrollable internal diseases (including unstable angina, myocardial infarction and cerebrovascular accident that occurred within 6 months). Patients who were unable to receive general anesthesia or surgical treatment due to conditions of other organs.

### 2.4. Mediastinal lymphadenectomy

The patients were placed in the supine position and the operation was performed through the ventral esophageal hiatus. Initially, an opening of the esophageal hiatus and an incision of both sides on the diaphragmatic feet at 1 to 2 cm were made. Subsequently, the surrounding tissue was dissociated, which was anterior to the pericardium, posterior to the aorta, lateral to the left and right pleura and upward to the inferior pulmonary vein. Thirdly, at the low margin of the inferior pulmonary vein, the tissue was stripped down along to the esophageal wall to the precut line and the lymphatic connective tissue around the esophageal area was removed completely. Finally, the inferior mediastinal LND was completed.

### 2.5. Observatory indicators

The clinical characteristics and the histopathological data of the enrolled Siewert II/III AEG patients were recorded, including gender, age, tumor site, tumor size, gross type, histological classification, infiltration depth, surgical approach, intravascular cancer embolus and tumor deposit. According to the age group proposed by the WHO, the patients were divided into the age < 60 years group and the age ≥ 60 years group. Tumor size was referred to the maximum diameter of the tumors. The gross type was divided into protruded type, flat type and depressed type according to the Japanese Gastric Cancer Guidelines (2014). Histological classification was divided into differentiated type (well-and-moderate differentiated adenocarcinoma, tubular adenocarcinoma) and undifferentiated type (poor differentiated adenocarcinoma, mucinous adenocarcinoma and signet-ring cell carcinoma). Tumor deposits are defined as nodules formed by clusters of tumor cells that exist independently within the drainage area of the tumor, without identifiable blood vessels, nerve fibers, lymphatic vessels, or lymph nodes, and regardless of the shape, size, and contour of the nodules.^[16]^

### 2.6. Follow-up

The patients were followed up every 6 months after surgery. After discharge, the patients were followed up by means of outpatient visit, telephone or mail. During the follow-up period, the patients received physical examination, laboratory tests, chest and abdominal CT scan assessment and annual endoscopic examination. The survival time was estimated from the operation date to the death date or until November 2023. The survival rate was determined by dividing the number of patients who survived for 3 years by the total number of enrolled patients.

### 2.7. Statistical analysis

Statistical analysis was performed using the SPSS 26.0 software. The count data were expressed as frequency and percentages and were compared using the *χ^2^* test, corrective *χ^2^* test or Fisher exact test. Ranked data were expressed as frequency and percentages and were compared using the rank sum test. Multivariate analyses were performed using the logistic regression method. Survival analyses were performed using the Cox regression. A *P* value <0.05 (*P* < .05) was considered for significant differences.

## 3. Results

### 3.1. Baseline data and clinicopathological characteristics

A total of 239 Siewert type II/III AEG patients, including 204 men (85.4%) and 35 women (14.6%), with a male-to-female ratio of 5.8:1 were included in the study. The patient age ranged from 27 to 83 years and the mean age was 63.1 years. A total of 77 cases out of 239 patients were < 60 years old (32.2%), whereas 162 cases were ≥ 60 years old (67.8%). A total of 165 patients suffered from Siewert II AEG, whereas 74 patients suffered from Siewert III AEG. Among them, 107 patients were combined with lymphatic metastasis and the remaining 132 patients did not present with lymphatic metastasis. No patients suffered from distant metastasis (Table 1).

**Table 1:** Correlation between clinicopathological factors and lymphatic metastasis in 239 patients of Siewert II/III AEG.

Clinicopathologic factors	Number	Lymphatic metastasis [n(%)]	No lymphatic metastasis [n(%)]	*P* value
Age <60 ≥60	77 162	39 (49.4%) 74 (45.7%)	38 (50.6%) 88 (54.3%)	.472
Gender M F	204 35	98 (48.0%) 15 (42.3%)	106 (52.0%) 20 (57.7%)	.570
Tumor site Siewert II Siewert III	165 74	81 (49.1%) 32 (43.2%)	84 (50.9%) 42 (56.8%)	.402
Tumor size <2 cm ≥2 cm	47 192	8 (17.0%) 105 (54.7%)	39 (83.0%) 87 (45.3%)	.000
Gross type Protruded Flat Depressed	31 16 192	11 (35.5%) 2 (12.5%) 100 (52.1%)	20 (64.5%) 14 (87.5%) 92 (47.9%)	.004
Surgical approach Proximal gastrectomy Total gastrectomy	109 130	39 (35.8%) 74 (56.9%)	70 (64.2%) 56 (43.1%)	.001
Infiltration depth T_is_ T_1_ T_2_ T_3_ T_4_	3 70 33 66 67	0 (0.0%) 8 (11.4%) 10 (30.3%) 38 (57.6%) 57 (85.1%)	3 (100%) 62 (88.6%) 23 (69.7%) 28 (42.4%) 10 (14.9%)	.000
Histological classification Differentiated Undifferentiated	114 125	36 (31.6%) 77 (61.6%)	78 (68.4%) 48 (38.4%)	.000
Intravascular cancer embolus Negative Positive	135 104	37 (27.4%) 76 (73.1%)	98 (72.6%) 28 (26.9%)	.000
Tumor deposit Negative Positive	224 15	99 (44.2%) 14 (93.3%)	125 (55.8%) 1 (6.7%)	.000

AEG = adenocarcinoma of esophagogastric junction.

### 3.2. Pattern of lymphatic metastasis

**Figure 1.**
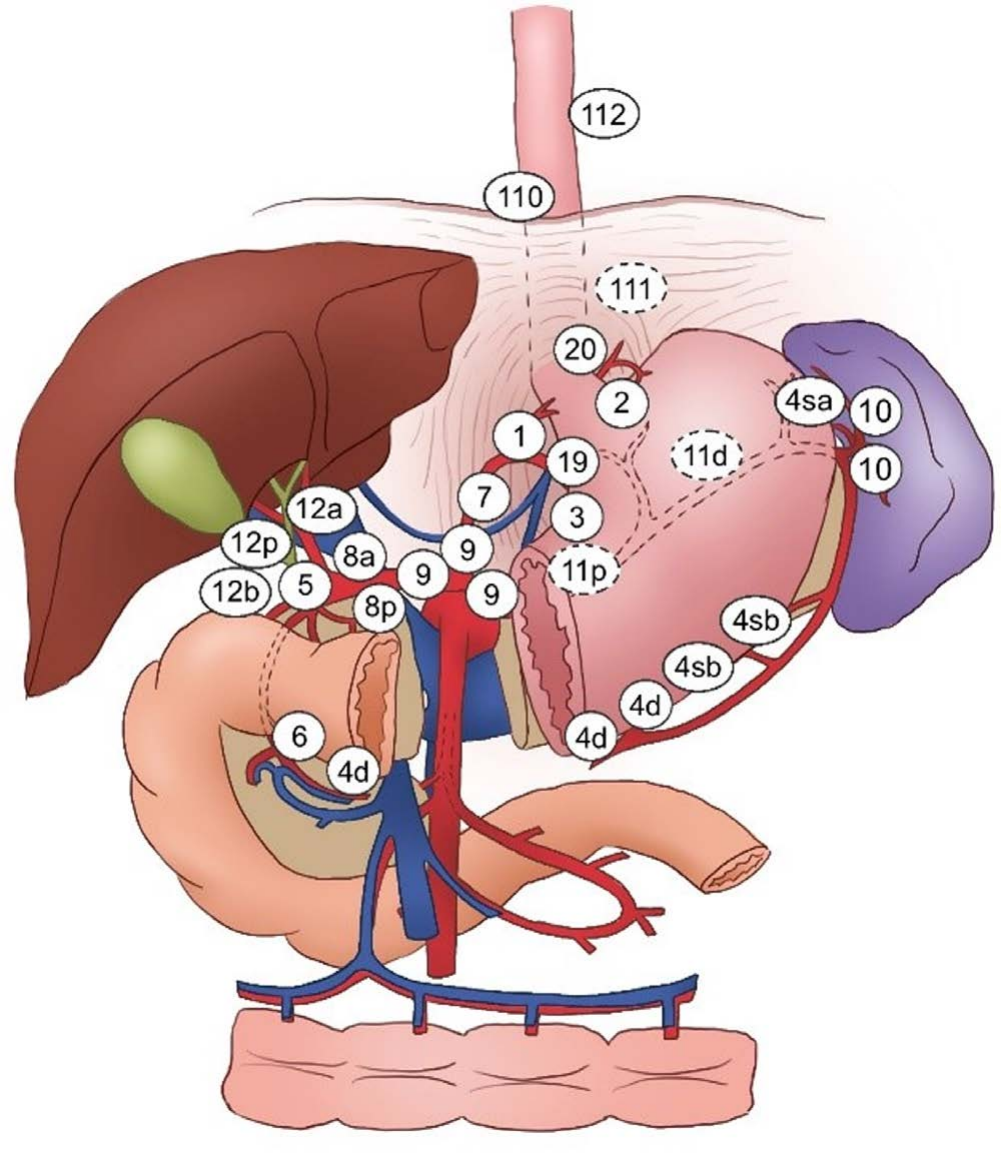
The locations of lymph nodes in each group. No.1 - Right cardial lymph nodes; No.2 - Left cardial lymph nodes; No.3 - Lesser curvature lymph nodes; No.4sa - Short gastric artery lymph nodes; No.4sb - Left gastroepiploic artery lymph nodes; No.4d - Right gastroepiploic artery lymph nodes; No.5 - Suprapyloric lymph nodes; No.6 - Infrapyloric lymph nodes; No.7 - Left gastric artery lymph nodes; No.8a - Anterior hepatic artery lymph nodes; No.8p - Posterior hepatic artery lymph nodes; No.9 - Para-aortic lymph nodes; No.10 - Splenic hilum lymph nodes; No.11p - Proximal splenic artery lymph nodes; No.11d - Distal splenic artery lymph nodes; No.12a - Lymph nodes along the hepatic artery within the hepatoduodenal ligament; No.12b - Lymph nodes along the bile duct within the hepatoduodenal ligament; No.12p - Lymph nodes posterior to the portal vein within the hepatoduodenal ligament; No.19 - Subdiaphragmatic lymph nodes; No.20 - Diaphragmatic hiatus lymph nodes; No.110 - Lower mediastinal lymph nodes; No.111 - Supradiaphragmatic lymph nodes; No.112 - Posterior mediastinal lymph nodes.

According to the National Comprehensive Cancer Network guidelines (2^nd^ edition, 2019), No. 1, 2, 3, 10, 7, 11 and 4 indicated higher LNM rate in the abdominal cavity of Siewert II AEG, whereas the metastatic rates for these cases were 36.13%, 28.57%, 27.78%, 25.00%, 18.87%, 13.89% and 11.76%, respectively (Table 2). Moreover, patients with Siewert II AEG included mediastinal lymph nodes that were prone to metastasize (No.110 and No. 111, Table 3), with a metastatic rate of 5.45% and 3.64%, respectively. In contrast to these observations, the pyloric lymph node, such as No.5 and No.6, rarely exhibited LNM (Table 2). No.5 (6.25%) and No.6 (8.33%) lymph nodes exhibited higher LNM rates in Siewert III AEG patients, while mediastinal LNM was not found in 74 patients compared with those of the Siewert II AEG patients. (Table 3). The locations of lymph nodes in each group are shown in Figure 1.

**Table 2:** Abdominal lymph node metastasis rate in Siewert II and Siewert III AEG patients.

Lymph node group	Siewert II (%, n/N)	Siewert III (%, n/N)	Total lymph node metastasis rate (%, n/N)
No.1	36.13% (43/119)	20.31% (13/64)	30.60% (56/183)
No.2	28.57% (26/91)	24.49% (12/49)	27.14% (38/140)
No.3	27.78% (35/126)	26.23% (16/61)	27.27% (51/187)
No.4	11.76% (12/102)	8.77% (5/57)	10.69% (17/159)
No.5	3.41% (3/88)	6.25% (2/32)	4.17% (5/120)
No.6	2.99% (2/67)	8.33% (3/36)	4.85% (5/103)
No.7	18.87% (20/106)	12.73% (7/55)	16.77% (27/161)
No.8	8.65% (9/104)	11.11% (6/54)	9.49% (15/158)
No.9	9.41% (8/85)	10.91% (6/55)	10.00% (14/140)
No.10	25.00% (5/20)	22.22% (2/9)	24.14% (7/29)
No.11	13.89% (10/72)	15.22% (7/46)	14.41% (17/118)
No.12	2.17% (1/46)	3.45% (1/29)	2.67% (2/75)
No.19	0% (0/5)	0% (0/5)	0% (0/10)
No.20	0% (0/12)	0% (0/6)	0% (0/18)

AEG = adenocarcinoma of esophagogastric junction.

**Table 3:** Mediastinal lymph node metastatic rate in patients with Siewert II/III AEG.

Lymph node grouping	Siewert II (%, n/N)	Siewert III (%, n/N)
No.110	5.45% (9/165)	0 % (0/74)
No.111	3.64% (6/165)	0% (0/74)
No.112	0.61% (1/165)	0% (0/74)

AEG = adenocarcinoma of esophagogastric junction.

### 3.3. Univariate analysis

Among the 239 Siewert II/III AEG patients included in the present study, 107 cases suffered from LNM with a metastatic rate of 45%. Of these, the metastatic rates were 17% and 54.7% in patients with a tumor size of < 2cm and ≥ 2 cm, respectively. This suggested that patients with a tumor size of ≥ 2 cm were more prone to LNM (*P* = .000). In addition, the LNM rate in patients with differentiated tumors was 31.6%, which was lower than that of 61.6% in patients with undifferentiated tumors (*P* = .000), whereas the LNM rate was elevated following an increase in the tumor infiltration depth (*P* = .000). The LNM rates in patients with intravascular cancer embolus and without intravascular cancer embolus were 73.1% and 27.4%, respectively (*P* = .000). Moreover, the parameters gross type (*P* = .004), surgical approaches (*P* = .001) and tumor deposits (*P* = .000) were all significantly different between these 2 groups (Table 1). However, the parameters age, gender, tumor site were not associated with LNM (Table 1).

### 3.4. Multivariate analysis

As shown in Table 4, the parameters infiltration depth, tumor deposit and intravascular cancer embolus were independent risk factors of LNM in Siewert II/III AEG patients. The LNM rate of advanced tumors was 0.038 times higher than that of the early tumor (95%CI: 0.011–0.139). Patients with tumor deposits exhibited a LNM risk that was 0.101 times higher than those without tumor deposits (95% CI: 0.011–0.904). The risk of LNM in patients with intravascular cancer embolus was 0.234 times higher than that of patients without intravascular cancer embolus (95%CI: 0.108–0.507) (Table 4).

**Table 4:** Multivariate analysis results for risk factors of lymph node metastasis in type II/III AEG patients.

Index	B	S.E	Walds	*P*	OR	95%*CI*
Infiltration depth Tumor deposit Intravascular cancer embolus	−3.263 −2.293 −1.451	0.656 1.118 0.394	24.703 4.205 13.574	.000 .040 .000	0.038 0.101 0.234	0.011–0.139 0.011–0.904 0.108–0.507
Constant	3.495	1.344	6.760	.009	32.950	-

AEG = adenocarcinoma of esophagogastric junction.

### 3.5. Follow-up and survival analysis

**Figure 2.**
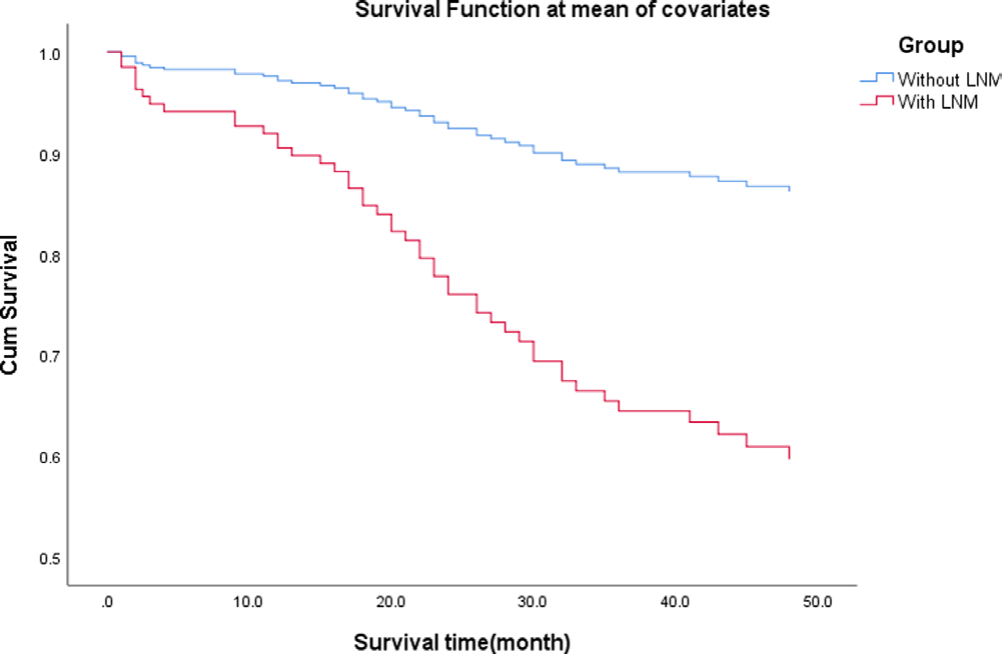
The 3-yr OS rate of AEG patients without LNM and with LNM was 88.0% and 64.5%, respectively (*P* = .001). AEG = adenocarcinoma of esophagogastric junction, LNM = lymph node metastasis, OS = overall survival.

**Figure 3.**
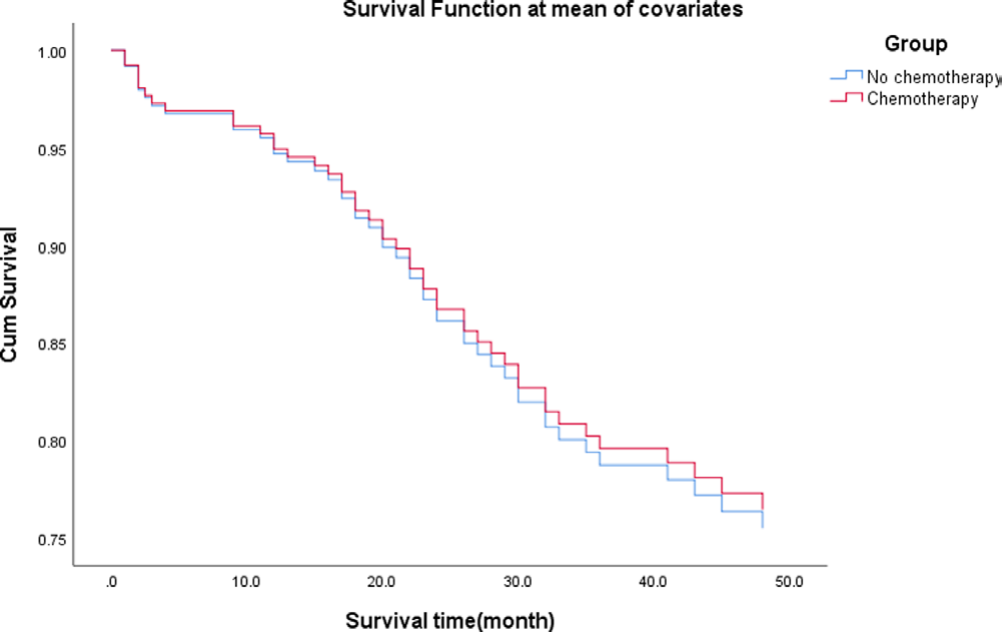
The 3-yr OS rate of AEG patients who accepted chemotherapy was higher than those who did not accept chemotherapy (*P* = .909). AEG = adenocarcinoma of esophagogastric junction.

The follow-up time of the 239 AEG patients was 1 to 119 months, whereas the median interval follow-up time was 45.1 months. During the follow-up period, 26 patients were lost to follow-up. The loss to follow-up rate was 10.9%. Among the 239 patients, 46 cases did not survive due to postoperative complications (7 patients), recurrence or distant metastasis (39 patients). Of these patients, 112 cases were followed up for more than 3 years. The median interval follow-up time period was 68.3 months, of which, the 3-year overall survival (OS) rate of the AEG patients without LNM (109 cases) and with LNM (104 cases) were 88.0% and 64.5%, respectively (*P* = .001, Fig. 2). In addition, the 3-year OS rate of AEG patients who accepted chemotherapy was higher than that of those who did not accept chemotherapy, although the difference was not statistically significant. (*P* = .909, Fig. 3).

## 4. Discussion

LNM is one of the most important prognostic factors for AEG.^[18]^ In addition, AEG can cause LNM and hematogenous metastasis at an early stage. Hence, the long-term prognosis of AEG patients has been demonstrated to be poor.^[19]^ Radical surgery has been proposed as the effective treatment for AEG, including complete resection of lesions and LND.^[20,21]^ However, Siewert II/III AEG is characterized by specific anatomical location and complex lymphatic drainage direction. Therefore, the correct evaluation of the LNM can determine the extent of optimal LND. The development of a reasonable treatment protocol is of great significance for improving the long-term prognosis in lymphatic metastasis patients with II/III AEG.

Certain studies have reported that the rates of mediastinal LNM are estimated from 5% to 25%,^[11,13,22,23]^ whereas the recurrence rates of mediastinal LNM following surgery ranged from 0 to 11%.^[12,22,24,25]^ In the present study, Siewert II AEG was associated with celiac and thoracic LNM. Metastasis to the celiac lymph nodes was noted, such as in the cases of No. 1, 2, 3, 10, 7, 11 and 4. In addition, the rate of mediastinal lymph nodes, such as that for No. 110 and No. 111, ranged from 5.45% to 3.64%. Particular attention has to be paid in order to perform both celiac and thoracic lymphadenectomy. However, in view of the low LNM rate of pyloric lymph nodes (NO.5 and No.6),^[26]^ tumor resection may be considered for this type of patients. Yamashita et al further showed similar results in a previous retrospective study.^[27]^ Siewert III AEG patients did not present with mediastinal LNM and presented with tumor recurrence in the thoracic cavity. It should be noted that the mediastinal LND can increase surgical trauma and cardiopulmonary complication, notably in the elderly population.^[28]^ Therefore, mediastinal LND is not necessary for Siewert III AEG patients. However, No.5 and No.6 LNM rates were estimated to 6.25% and 8.33%, respectively. A previous study reported that the dissection of pyloric lymph nodes could improve long-term prognosis for Siewert III AEG.^[26,29]^ Therefore, this type of patients should be treated with dissection of the pyloric lymph nodes. Moreover, we should emphasize on the en-bloc resection and avoid fragmented resection.^[30]^

The assessment of the risk of LNM can aid the selection of the appropriate extent of LND and individualized treatment strategy. At present, there is no effective method to evaluate LNM prior to surgery. However, the clinicopathological data exert a favorable prediction effect. In the present study, the infiltration depth, tumor deposit and intravascular cancer embolus were independent risk factors for LNM in patients with Siewert II/III AEG. Previous studies have reported that the LNM rate was significantly increased following an increase in the tumor infiltration.^[31]^ The present study indicated similar outcomes demonstrating that the LNM rate was increased from 0 to 85.1% when the tumor infiltrated deeper gradually. This outcome may be attributed to the abundance of lymphatic capillaries in the submucosa, resulting in LNM.^[32,33]^ In addition, the tumor deposits are currently part of the TNM staging system for colorectal cancer.^[16]^ Chen Hao et al revealed that patients with positive tumor deposits have significantly worse prognosis than those with negative tumor deposits.^[34]^ Research has shown that tumor deposits are common in gastric cancer and are an indicator of invasive characteristics and a strong and independent prognostic factor, which should be included in the gastric cancer staging strategy.^[35]^ Although the clinical significance of TDs is recognized, the exact mechanism of their development remains unclear. One hypothesis suggests that TDs occur during the process of LNM, referred to as “in-transit metastasis.”^[36]^ This finding is similar to our study, which identified tumor deposit as an independent risk factor influencing LNM. However, we need to further verify these findings using large-sample data. In addition, among the 104 patients who were combined with intravascular cancer embolus, 76 patients exhibited LNM. The incidence was higher than those patients who did not present with intravascular cancer embolus. A previous study demonstrated that 54 gastric cancer patients with intravascular cancer embolus exhibited 51 cases with LNM (94.4%).^[33]^ This may be due to the capillary wall that does not contain a basal membrane and is composed of endothelial cells. The majority of these cells are irregularly arranged. Therefore, the capillary wall has greater permeability than the capillary and is more susceptible to cancer cell invasion.^[33]^ Nevertheless, since it is difficult to determine whether patients present with intravascular cancer embolus or tumor deposits preoperatively, tumor infiltration depth remains valuable indicator for the prognosis of the disease.

In the present study, survival analysis revealed that LNM exhibited significant differences in the 3-year overall survival rate. The data indicated that patients with LNM exhibited worse long-term prognosis. It was reported that proximal gastric cancer could develop LNM and exhibit early recurrence.^[37]^ Kim et al revealed that the survival rate was decreased following an increase in the LNM rate.^[38]^ The data indicated that LNM was an independent prognostic risk factor for 86 patients.^[39]^ A study conducted in China revealed that the 3-year and 5-year survival rates of 231 gastric cancer patients without LNM were 69.7% and 63%, respectively, while the survival rates of 481 gastric cancer patients with LNM were 38.4% and 28.7%, respectively (*P* = .001).^[40]^ In addition, a multi-center study demonstrated that Siewert II AEG patients with mediastinal LNM exhibited a higher recurrence rate and a poor prognosis.^[12]^ As a result, LNM should be considered a prognostic evaluation index for gastric cancer patients who have received radical operation.

However, perioperative chemotherapy can improve the long-term prognosis of AEG patients to some extent. The current result was consistent with previous studies.^[41]^ Results from the MAGIC trial showed that the AEG patients who received perioperative chemotherapy had better survival rates and lower rates of cancer recurrence than those who only had surgery.^[42]^ The FOLFOX treatment appears to have a significant activity as first-line treatment for advanced gastric cancer patients, with an encouraging response rate and a mild toxicity profile.^[43]^ The EORTC40954 clinical trial indicated that the preoperative chemotherapy combined with surgery could increase R0 resection but failed to show survival benefit in stage III and IV (cM0) of AEG or gastric cancer patients.^[44]^ In addition, the FNCLCC and FFCD9703 clinical trials revealed that perioperative chemotherapy could improve the curative surgical rate, overall survival (OS) and the disease-free survival.^[45]^ In 2011, the ACTS-GC trial reported that postoperative treatment with S-1 chemotherapy could prolong the 5-year OS and disease-free survival.^[46]^ The CLASSIC study for stage II to stage IIB gastric cancer revealed that patients who received chemotherapy following surgery exhibited optimal prognosis than those treated with surgery alone.^[47]^ Recently, the POET trial compared chemotherapy and surgery with induction chemotherapy, chemoradiotherapy and surgery.^[48]^ The data demonstrated that induction chemotherapy and chemoradiotherapy could prolong progression-free survival.^[48]^ The present study demonstrates that AEG patients who received chemotherapy had a higher 3-year survival rate compared to those who did not receive chemotherapy. The lack of statistical significance in the observed differences may be attributed to several factors, including the retrospective nature of this single-center study, the relatively small sample size and suboptimal selection of perioperative chemotherapy regimens. Therefore, chemotherapy has become a necessary treatment modality in advanced AEG.

The present study exhibits certain limitations. It is a retrospective, single-center study, involving a small number of samples. Therefore, the results are unlikely to fully reflect the regulation of LNM and long-term prognosis. There may be certain bias in the analysis of the clinical data. Hence, we need to further confirm the current findings by large-sample, randomized controlled studies.

## 5. Conclusions

In summary, LNM in patients with Siewert II/III AEG was mainly associated with the parameters infiltration depth, tumor deposit and intravascular cancer embolus. For Siewert II AEG patients, it is reasonable to excise both celiac and mediastinal lymph nodes, notably for No.1, 2, 3, 4, 7, 10, 11, 110 and No.111 lymph nodes. However, pyloric lymph nodes did not require dissection. For Siewert III AEG patients, the mediastinal LND was not required. However, it is necessary to perform celiac lymphadenectomy, including pyloric lymph nodes. Moreover, patients with LNM exhibited worse long-term prognosis. Perioperative chemotherapy can improve the prognosis of AEG patients. All the results remain to be further verified using large-sample, multi-center, randomized controlled studies.

## Acknowledgments

We wish to thank MedSci Company for careful proof-editing of this manuscript.

## Author contributions

**Conceptualization:** Jie Yin.

**Formal analysis:** Yidong Huang.

**Funding acquisition:** Jun Zhang, Guangyong Chen.

**Investigation:** Guangyong Chen.

**Methodology:** Yidong Huang.

**Project administration:** Rui Xu.

**Software:** Rui Xu.

**Supervision:** Jie Yin, Xiaoye Liu, Jun Zhang, Guangyong Chen, Zhongtao Zhang.

**Writing – original draft:** Zhi Zheng, Yidong Huang, Haiqiao Zhang.

**Writing – review & editing:** Jun Zhang.
